# Linking land cover and species distribution models to project potential ranges of malaria vectors: an example using *Anopheles arabiensis* in Sudan and Upper Egypt

**DOI:** 10.1186/1475-2875-11-264

**Published:** 2012-08-06

**Authors:** Douglas O Fuller, Michael S Parenti, Ali N Hassan, John C Beier

**Affiliations:** 1 Department of Geography and Regional Studies, University of Miami, Coral Gables, FL 33124-2221, USA; 2 GIS Division, McGinley and Associates Inc., Las Vegas, NV, 89118, USA; 3 Department of Basic Environmental Sciences, Institute of Environmental Studies & Research, Ain Shams University, Cairo, Egypt; 4 Department of Epidemiology and Public Health, University of Miami Miller School of Medicine, Miami, FL, 33136, USA

**Keywords:** *Anopheles arabiensis*, Sudan and Upper Egypt, Species invasion potential, Irrigation, Land change Modeler, MaxEnt

## Abstract

**Background:**

*Anopheles arabiensis* is a particularly opportunistic feeder and efficient vector of *Plasmodium falciparum* in Africa and may invade areas outside its normal range, including areas separated by expanses of barren desert. The purpose of this paper is to demonstrate how spatial models can project future irrigated cropland and potential, new suitable habitat for vectors such as *An. arabiensis*.

**Methods:**

Two different but complementary spatial models were linked to demonstrate their synergy for assessing re-invasion potential of *An. arabiensis* into Upper Egypt as a function of irrigated cropland expansion by 2050. The first model (The Land Change Modeler) was used to simulate changes in irrigated cropland using a Markov Chain approach, while the second model (MaxEnt) uses species occurrence points, land cover and other environmental layers to project probability of species presence. Two basic change scenarios were analysed, one involving a more conservative business-as-usual (BAU) assumption and second with a high probability of desert-to-cropland transition (Green Nile) to assess a broad range of potential outcomes by 2050.

**Results:**

The results reveal a difference of 82,000 sq km in potential *An. arabiensis* range between the BAU and Green Nile scenarios. The BAU scenario revealed a highly fragmented set of small, potential habitat patches separated by relatively large distances (maximum distance = 64.02 km, mean = 12.72 km, SD = 9.92), while the Green Nile scenario produced a landscape characterized by large patches separated by relatively shorter gaps (maximum distance = 49.38, km, mean = 4.51 km, SD = 7.89) that may be bridged by the vector.

**Conclusions:**

This study provides a first demonstration of how land change and species distribution models may be linked to project potential changes in vector habitat distribution and invasion potential. While gaps between potential habitat patches remained large in the Green Nile scenario, the models reveal large areas of future habitat connectivity that may facilitate the re-invasion of *An. arabiensis* from Sudan into Upper Egypt. The methods used are broadly applicable to other land cover changes as they influence vector distribution, particularly those related to tropical deforestation and urbanization processes.

## Background

Unintentional introductions of mosquito vectors into environmentally suitable areas outside their normal ranges have led to establishment of breeding populations and subsequent outbreaks of human malaria and other important mosquito-borne diseases [1-7]. Establishment of founder populations depends on many variables, including abiotic similarity between source and invaded sites, absence of predators and parasites that may limit vector populations within their normal ranges, lack of rigorous control measures within invaded territories and sufficient sources of blood meals. In addition, mosquito invasion events have been facilitated by human migration, global trade, and international travel and tourism [7,8]. While much recent literature has focused on the spread of invasive species, such as *Aedes aegyptiAedes albopictusAedes atropalpus* that breed in containers, climate change coupled with ecosystem disturbance may also favour the spread of various anophelines to areas where malaria has been eliminated or was previously absent [5,9]. Specific invasion events associated with notable outbreaks of malaria include the long-distance dispersal of *Anopheles gambiae* from West Africa to north-east Brazil in 1930, which over a nine-year period produced a major increase in human malaria cases having an estimated 20-25% death rate [1,6]. Further, in 1943 a major malaria epidemic occurred in Egypt associated with the spread of *An. arabiensis* (a member of the *An. gambiae* species complex) from Sudan along the Nile Valley [1,10]. This particular outbreak produced some 130,000 deaths within a two-year period until successful control and vector elimination measures were implemented in late 1944 [1]. At that time, the limits of the infestation were known and confined to irrigated areas well to the north of the current study area in Asyut Governorate. This facilitated efficient application of larvicidal agents that were used in the successful eradication campaign [1].

Although rainfall and temperature exert critical controls over the life cycles of both mosquitoes and Plasmodium, changes in land use and land cover may also facilitate (or prevent) the spread of malaria vectors [11-14]. Deforestation in tropical lowland (<500 m a s l) environments has received emphasis in the recent literature, particularly associated with frontier malaria in Latin America where forest has been converted to pasture or farmland. For example, numerous studies have established a link between deforestation and abundance of *Anopheles darlingi*, which is one of the most important malaria vectors found near tropical forest fringes in the Neotropics [12,15-17]. Irrigation in highly seasonal or arid and semi-arid environments also facilitates establishment and spread of malaria vectors [13,18,19]. Patz *et al.*[13] postulate that the development of irrigation and other human-induced changes in surface hydrology that result in slow-moving water are likely to be more significant for vector breeding in regions where malaria is either absent or hypo-endemic, such as in North Africa or India. Over the past several decades irrigated cropland has greatly increased in the Middle East and North Africa, with irrigated area covering an estimated 26 million hectares (ha) in 1998. By 2030 irrigated area in the region is projected to increase by 25% to meet local and global food demand [20]. Therefore, it is reasonable that future irrigation development will pose a major risk of malaria outbreaks as vectors may disperse from source regions to newly irrigated areas.

Opportunities exist for gaining a more comprehensive understanding of the interactions between environmental change and vector invasion potential using different types of space-time models that can simulate environmental change or species distribution [21,22]. Such models can take into account key factors that help to increase or isolate vector populations such as climate, physical barriers or corridors such as large rivers, lakes or seas, mountains, vegetation type and distance between suitable habitats [10]. While species distribution models (SDMs) have provided new insights into areas where malaria vectors are likely to encounter suitable habitats, most SDM applications for mapping malaria vectors have emphasized climate as the principal factor that controls potential habitat suitability [18,21-25]. However, some SDMs can accommodate land cover information in discrete form (i.e. land cover classes) and this creates the possibility to link land change models (LCMs) with SDMs to map potential vector distribution driven by anthropogenic changes such as urbanization, irrigation and deforestation. This study demonstrates how LCMs and SDMs can be linked to project future changes in the potential range of *An. arabiensis* associated with expansion of irrigated cropland in the Nile Valley of Sudan and Upper Egypt.

*Anopheles arabiensis* was selected as a case study because authorities at the Ministry of Health in Egypt are particularly concerned that this efficient vector species may re-invade Egypt from the Sudan and cause wide-scale epidemics of *Plasmodium falciparum,* as it did in 1943. Concern is also justified given the development of new transportation and water management initiatives in Northern State, Sudan and downriver within the Nile Valley in Upper Egypt [10,26]. In addition, agriculture in the region is mainly based on irrigation from Nile waters and many people live alongside the river where land is often used for cultivation and grazing, thus creating a potential corridor for dispersal. Climate change predictions based on outputs from General Circulation Models (GCMs) and hydrological models suggest that most of North Africa will experience progressively drier conditions in the latter part of this century [27-29]. While the flow of the Nile is influenced primarily by rainfall and water management upstream, coupled GCM-hydrological models indicate that stream flow at Aswan is likely to decline from 2040–2100 and, therefore, water capture, diversion and irrigation are likely to become even more important strategies to ensure adequate food production in Egypt and Sudan [29]. While the climate literature lacks specificity about future surface wind directions and speed associated with warming in the 21^st^ century, increased variability and storm events associated with atmospheric warming [28] may enhance opportunities for long-distance dispersal of the vector.

**Figure 1:**
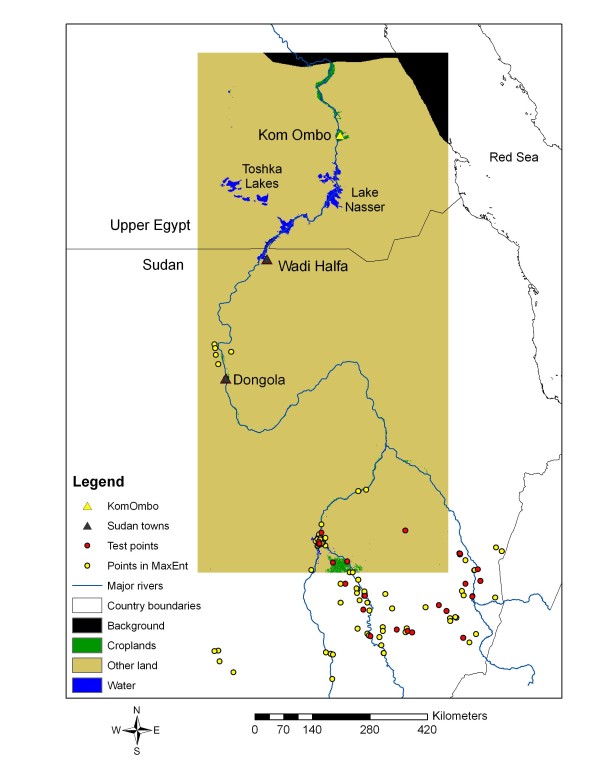
**2001 land cover with occurrence points shown.** The rectangle shows the main study area where projected habitat patches were analysed and the black background indicates areas either outside the original MODIS imagery or the Red Sea which were not considered in the analysis.

*Anopheles arabiensis* is particularly adaptive to environmental change and is known to have a wide range of feeding and resting patterns and can adapt quickly to control measures such as indoor residual spraying [30]. Its larval habitats are similar to those of *An. gambiae* although *An. arabiensis* is able to use a wide range of aquatic habitats, including slow flowing, partially shaded streams and a variety of large and small natural and man-made habitats [30]. The northern edge of the distribution of *An. arabiensis* is downriver beyond Dongola, approximately 300 km south of the border with Egypt, but occasional incursions of the species have been found as far north as Wadi Halfa [10] (Figure1). The main hypothesis of this investigation is that the re-invasion of *An. arabiensis* has been limited by wide expanses of hyperarid desert, low human population density, and limited human movement between Egypt and Sudan. In addition, the Ministries of Health in both Egypt and Sudan conduct annual surveillance in the border area and intensively spray with residual insecticides potential breeding habitats and dwellings, which may also limit dispersal from south to north. However, expansion of densely irrigated cropland from Lake Nasser, the Toshka Lakes and Kom Ombo northward may provide foci for establishment and future range expansion (Figure1). 

## Methods

### Model descriptions

Two well-known models were selected for analysis: the Land Change Modeler (LCM), which was used for projecting future changes in irrigated cropland extent, and MaxEnt, for projecting future changes in potential *An. arabiensis* distribution. The LCM is available either as an extension to the popular ArcGIS software or as a module within Idrisi, a widely used raster-based GIS package. Land cover projections of the LCM were fed into MaxEnt to project a range of potential distributions. The LCM employs a set of spatial data layers associated with land cover changes, such as rivers, roads, settlements, which distinguishes it from agent-based models, which are generally based on behavioural aspects of agents of change and attempt to model decisions often made at the local level [22,31,32]. Consistent with other studies that address future environmental conditions in North Africa [20,28,29], the year 2050 was selected as the endpoint of the simulations using both a business-as-usual (BAU) and a Green Nile Scenario, which assumes high probability of desert land changing to irrigated cropland (details below). LCM establishes the quantity of change by evaluating the empirical Markov matrix based on comparison between the initial and second land cover maps in time and then assumes this same transition probability as it projects into the future [33]. The BAU scenario simply assumes that transition probabilities derived from past changes remain constant through time and are derived from the changes observed between two land cover maps. The LCM allows users to vary the transition probabilities and so the transition probabilities were modified for a set of LCM runs to examine how increasing the probability of land changing from desert to irrigated cropland would change spatial allocation (i.e. the Green Nile scenario) of irrigated cropland, which is clearly more suitable habitat for this vector species than barren desert. For the LCM simulations, a neural network approach was selected to develop a transition probability layer based on prior experience with this particular algorithm in the context of land change modeling [34].

Although many SDMs have been developed, MaxEnt was selected since it is robust, it accepts categorical data, and it produces accuracies that typically compare with or exceed other SDMs [35,36]. This SDM uses a machine-learning approach with a set of user-specified covariates that include species occurrence points, used to develop a set of training samples, and spatial layers representing environmental variables (generally denoted as **z**) to produce predicted probability surfaces (denoted here as p(***s***)) in raster format. The model employs a maximum entropy approach that integrates model covariate selection and controls for overfitting by using smoothing and identifies how the covariates contribute to the model. The model minimizes relative entropy, a measure of dispersion or uncertainty associated with a random variable, through a Gibbs distribution, which is an exponential family model:

(1) $$f_{1}\left(z\right)=f\left(z\right)e^{\;\left(z\right)}$$

where ***f***_1_(**z**) is the probability density of covariates across a landscape at known species locations and (**z**) = α + β·h(**z**). α is a normalizing constant that ensures ***f***_1_(**z**) sums to 1 and β is an estimated parameter that weighs the contribution of each covariate using a log likelihood approach [36]. For a complete explanation of MaxEnt, readers are referred to Elith *et al.*[37]. For all MaxEnt runs, the default settings were utilized as generally followed in other SDM intercomparison studies that involve this particular model [35].

### Application of the land change models and MaxEnt models

Two land cover maps from 2001 and 2009 were obtained, which were derived from classification of Moderate Resolution Imaging Spectroradiometer (MODIS) imagery produced as the 500 m MCD12Q1 data obtained from a NASA website [38]. The International Geosphere-Biosphere Programme (IGBP) land cover scheme was selected, which includes 17 classes, including two that contain croplands that were combined to form a single cropland class (Figure1). All other land cover types except water were reclassified to form a single class referred to as “other lands,” which was dominated by the barren or sparsely vegetated classes in the IGBP scheme. The study area shown in Figure1 was selected to cover the Nile River corridor north of the 200 mm rainfall line as determined from rasterized climate data (described below). Previous analysis of the MCD12Q1 product for the study area showed excellent agreement with Landsat imagery gridded to 30 m spatial resolution as well as MODIS 250 m imagery of the normalized difference vegetation index (NDVI), thus providing high confidence in the accuracy of the land cover maps used in the LCM [26]. Three distance layers from water bodies, major roads [39] and irrigated cropland were created as proximate drivers in the LCM.

In addition to land cover data, climate data was also used, including mean monthly minimum/maximum temperature and precipitation, obtained from the WorldClim database gridded to approximately 1 km resolution [40]. NDVI was also derived from MODIS imagery gridded to 1 km resolution and were also obtained for the period 2001–2010 from the same NASA website that supplied the MCD12Q1 products and a range of summary statistics was calculated including mean annual NDVI, annual range and standard deviation images. As NDVI provides a proxy measure of surface moisture, these images were also used in MaxEnt as environmental covariates. Table1 summarizes the covariates used in both the LCM and MaxEnt models. 

**Table 1:** List of covariates used in the Land Change Modeler and MaxEnt

**Spatial Model**	**Covariates/Drivers [Sources]**	**Derived Layer(s) Used in Model Projections**
Land Change Modeler (LCM)	Land cover classification 2001 [38]	Distance from water bodies
	Land cover classification 2009 [38]	Distance from irrigated cropland
	Major roads and rivers [39]	Distance from roads
MaxEnt	Mean monthly maximum and minimum temperature [40]	Current and future land cover projections from the LCM scenarios
	Mean monthly precipitation [40]	Annual range (maximum-minimum) of NDVI values
	Shuttle Radar Topography Mission (SRTM) [41]	
	*An. arabiensis* points [42]	
	16-day composites of the normalized difference vegetation index (NDVI), 2001–2009 [38]	

Sixty-four different MaxEnt experiments were conducted using different combinations of land cover, NDVI, climate layers and an elevation layer derived from the Shuttle Radar Topography Mission (SRTM) 90 m data [41]. This number of experiments provided a sufficient sample to evaluate model performance with different combinations of covariates. An experiment was defined as a unique set of environmental covariates in which different combinations of covariates were input to the model. Fifty of these experiments involved 2001 conditions to evaluate how the SDM performed for near-current conditions and the remaining experiments involved inclusion of the BAU and Green Nile projections. Species occurrence points were obtained from the Malaria Atlas Project (MAP) [42] and the points were gridded to 1 km resolution to match the resolution of the climate data. The points include collections of both immature and adult forms obtained since 1985, have a spatial precision of 10^-3^ degrees and are approximately concurrent with the NDVI and climate layers used in the analysis (Sinka, pers comm). Of the total 104 unique occurrence points that fell within the study area, 25 occurrence points were randomly selected to independently check the results of each model experiment (Figure1). According to the MAP database, most collections consisted of adults resting inside houses. Note that many of the points fell to the south of the Nile Corridor in Sudan, which was necessary to obtain a reliable statistical representation of the environmental conditions associated with presence of *An. arabiensis*. The independent test points were used to calculate statistics such as the mean p(***s***) and standard deviation for each model experiment to evaluate the accuracy of different experiments.

Several different bias layers were created to de-bias the data for unrepresentative (i.e. highly clustered) sampling of specimens. After a number of trials with different bias layers, a bias layer was selected based on examination of the point distribution in Figure1, which shows a clear tendency for collections near rivers, as well as informed assumptions based on the authors’ field survey experience; i.e. that the probability of sampling for *An. arabiensis* declines as a function of distance from major roads and coasts. To create this particular bias layer, a procedure developed by Fuller *et al.*[25] was applied. Thus, each experiment was evaluated both qualitatively by comparing the MaxEnt outputs with known distribution and by extracting probability values using the independent test points selected randomly from the data set.

The lowest presence threshold (LPT) method was used for setting thresholds to evaluate presence/absence from the MaxEnt experiments. This method uses the lowest predicted value associated with any one of the observed occurrence points and it can be interpreted ecologically as pixels predicted as being at least as suitable as those where a species presence has been recorded [43]. It is thus considered a highly conservative way to map the minimum predicted distribution. In addition, the LPT approach reduces omission error to zero in the training data set [43]. A 2050 “Green Nile” scenario was created by manipulating the Markov transition matrix such that the probability of a pixel classified as “other land” changing to a pixel classified as “cropland” was increased to 0.200, while no change (other land not changing to any other land cover category) was assigned a probability of 0.800. The same transition probability in the BAU scenario derived from overlay of the 2001 and 2009 MODIS land cover maps was 0.028, thus the Green Nile scenario assumed nearly an order of magnitude increase in probability of other land becoming this land cover type.

Land cover layers for 2001, the BAU and the Green Nile scenarios were then passed to the MaxEnt SDM to see how habitat suitability may change by 2050 as a function of potential changes in irrigated cropland. A Monte Carlo approach was used to simulate 2050 NDVI by taking the mean and standard deviation of 2009 NDVI range (maximum-minimum) extracted for irrigated lands (mean = 0.577, SD = 0.098). NDVI range was used because it provides a convenient summary statistic that relates to crop phenology. The NDVI distribution for irrigated cropland was estimated by evaluating the image histogram of 2009 NDVI values for irrigated land and by extracting 2,230 randomly sampled points that fell on irrigated cropland. The probability distribution was evaluated using the Chi Square statistic, which was significant for a normal distribution (Chi-Square = 57.46, DF = 4, p = 0.00). The resultant LPT-based maps were then analysed using a set of GIS operations to quantify key landscape characteristics including the number of suitable habitat patches, their area and distances between patches within a 25-km buffer around water bodies. This analysis served to provide basic information on the spatial configuration of projected cropland, including gaps, patch size, corridor length, etc., from which one may infer re-invasion potential through the Nile Valley under different scenarios. Such data may also be useful in guiding parameterization of new metapopulation models that consider habitat patch dynamics of malaria vectors in areas of low transmission [44].

## Results

For 2001 land cover experiments, mean probability of species presence, p(***s***), obtained from the test points, ranged from 0.27 for one experiment that included monthly minimum and maximum temperature only to 0.64 and those that included NDVI range, SRTM and land cover, respectively. The mean p(***s***) value for all experiments that included either monthly temperature or precipitation covariates was 0.51 (SD = 0.11), whereas the mean for experiments that included only land cover, NDVI or elevation was 0.57 (SD = 0.05). It was noted that experiments that included temperature generally produced anomalously high p(***s***) values in desert areas far from water bodies, which further suggests that climate data failed to enhance the model outputs. This may have been due to the uniformly hot, arid conditions that characterize northern Sudan and Upper Egypt.

**Figure 2:**
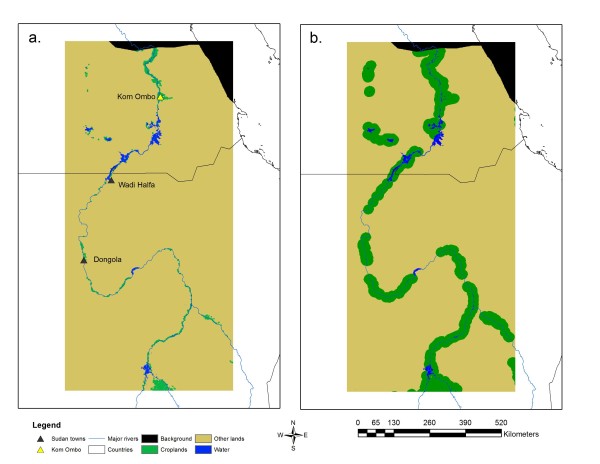
Land cover projections from the LCM: a. BAU scenario and b. Green Nile Scenario.

**Figure 3:**
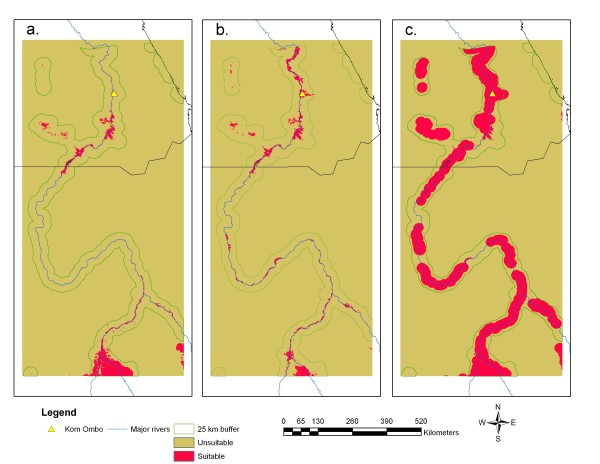
**Potential distribution of *Anopheles arabiensis *obtained from the SDM, MaxEnt including a. the 2001 distribution, b. BAU projection; c. Green Nile scenario.** The 25 km buffer around water bodies is also shown.

Figure2 shows the 2050 LCM outputs for both the BAU and Green Nile scenarios and reveals two extremes with only modest growth of irrigated cropland along the Nile noticeable relative to 2001 land cover shown in Figure1. This is probably a more realistic scenario compared to Figure2b, which represents an extreme case that would involve high investment in costly irrigation infrastructure and a high degree of political stability to realize even by mid-century. The projected distribution of irrigated cropland in this figure resembles the buffer around water bodies (Figure3) and shows how the LCM produced range expansion by adjacency effects along major fronts as opposed to expansion through long-distance dispersal via establishment at disjunct foci.

Figure3a reveals potential distribution in 2001 based on 2001 land cover, SRTM, and NDVI range (mean p(***s***) = 0.60, SD = 0.23, LPT = 0.23), whereas Figures 3b and 3c show the 2050 results when Figures 2a and 2b were input to the SDM. The LPT values were 0.45 and 0.29 for the BAU and Green Nile scenarios, respectively. All three projections reveal potentially suitable habitat around the Toshka Lakes, which have experienced modest expansion of irrigation and have been targeted by the Egyptian Government as an area for major agricultural development [26]. In addition, Figure3 reveals an area of potentially suitable habitat associated with Lake Nasser in Upper Egypt, which is consistent with understanding of the vector’s larval habitat characteristics [30]. The maps depicting future scenarios (Figures 3b and 3c) correspond closely to the distribution of irrigated cropland in the BAU and Green Nile scenarios. Figures 3b and 3c also show the strong influence that land cover may have on SDM projections. It is important to note that the LPT approach used to threshold these future projections implies that test points representing *An. arabiensis* habitat locations are static and that the species will remain present in these spots by 2050.

The results in Table2 reveal a range of possible outcomes by 2050 with an increase of nearly 82,000 sq km in potential *An. arabiensis* range expansion between the BAU and Green Nile scenarios, which equates to approximately 49% of the area within the 25 km buffer around water bodies (Figure3), which coincides approximately with the widest area of irrigated cultivation found around Kom Ombo [26]. Table2 also provides the patch characteristics of the three maps shown in Figure3. Interestingly, the BAU scenario (Figure3b) produced a more fragmented arrangement of potential habitat patches than the 2001 projection (Figure3a) with fewer smaller patches relative to 2001. This result may be an artefact of the decrease in potentially suitable habitat at the southern end of the study area. Specifically, the MODIS-based land cover maps show that in 2001 cropland covered 405,758 ha, whereas in 2009 the cropland area decreased to 382,341 ha. Thus, in the BAU scenario this would lead the Land Change Modeler to project a decrease in irrigated cropland area by 2050, which is consistent with the decrease shown in Table2. Figure3a also contains an apparent anomaly in that the area from Kom Ombo northward is excluded as potential habitat while the BAU and the Green Nile Scenarios identified these areas as potentially suitable, which appears reasonable given the density of irrigation present in this part of Upper Egypt [26]. The maximum distance between patches reported in Table2 is considered biologically significant as it indicates the largest desert gap that an adult *An. arabiensis* would have to cross to reach suitable habitat along the Nile corridor. These distances were 228.37, 64.02 and 49.38 km for Figures 3a-3c, respectively. Thus, despite the large increase in mean patch area and decrease in mean patch distance between the BAU and Green Nile scenarios, these model results suggest that significant gaps would still exist that may prove inimical to dispersing adult individuals of *An. arabiensis*, which typically move 350–650 m day^-1^ within suitable habitats [45]. Of course, the dispersal capacity for this species may be greatly facilitated through chance events related to downwind dispersal, human action, particularly transportation of eggs and immature forms through future transportation and trade linkages, which are planned for development [10,46]. It should be noted that the models used in this demonstration were not parameterized to account for growth of linear connections between patches although this may be possible in future scenario development. 

**Table 2:** Summary landscape statistics for the three projections of potential range

**Metric**	**2001**	**2050 BAU**	**2050 Green Nile**
Total area of suitable habitat in km^2^	15,484	11,633	93,646
Number of patches	315	324	35
Mean patch area in sq km (SD)	5,103.34 (3,993.26)	896.26 (653.14)	14,331.17 (7,886.03)
Mean inter-patch distance in km within 25 km buffer of water bodies (SD)	19.38 (27.65)	12.72 (9.92)	4.51 (7.89)
Maximum inter-patch distance (km) within 25 km buffer of water bodies	228.37	64.02	49.38

## Conclusions

This study provides a first demonstration of how land change and species distribution models may be linked to advance understanding of potential distributions of malaria vectors as a result of anthropogenically driven changes in land cover and use, in this case related to irrigation and agricultural expansion. With a few exceptions, the spatial projections of potentially suitable range (Figure3) generally produced results consistent with understanding of the bionomics of *An. arabiensis*. Figure3 also reveals three large gaps along the Nile corridor in Sudan where quarantine efforts may be targeted in the future. Further, the projected ranges for the three maps show potential habitat patches associated with flowing and man-made water bodies and sparsely vegetated areas within the Nile Valley, indicating the broad range of environments associated with the species [30]. Generally, such maps produced by linked LCM-SDMs are suitable for inferring potential re-invasion in so far as they allow quantification of distances from current or future occurrence sites, connectivity corridors and potentially suitable habitats of particular sizes that may be related to the flight range of this species as well as other vectors. Future validation of land cover change projections may be supported by use of moderate resolution satellite imagery such as MODIS 250 m images that clearly reveal irrigated patches of cropland. In addition, mosquito collection data from on-going and future surveillance efforts may also be employed to check the accuracy of potential range projections using a standard error matrix approach.

Although little is known about long-distance dispersal in *An. arabiensis*, many studies have demonstrated long-distance, wind-aided dispersal of adult female mosquitoes over distances ranging from tens to hundreds of km [46]. Thus, the distances between potential habitat patches shown in Table2 appear to be surmountable if chance establishment were to occur through the action of wind or human agents. Consistent with this conclusion, several reports indicate that anopheline vectors, including *Anopheles multicolorAnopheles sergentii* and *Anopheles algeriensis*, have already been found in the area around the Toshka Lakes and other areas experiencing land use and cover changes, such as along the Gulf of Suez,where the presence of *Anopheles pharoensis* has been recorded recently [47-49].

The results in this study should be considered as heuristic in that they serve to illustrate the potential of using LCM outputs to drive SDMs as a way to explore different potential distribution scenarios related to irrigation in arid environments. The outputs of SDMs driven by LCM projections may be connected further to mathematical models such as metapopulation models that consider patch size and connectivity to simulate colonization, extinction, as well as pathogen transmission and epidemic processes [44]. In addition, future applications of LCMs may include growth of linear connections, particularly establishment of new roads and irrigation canals that are likely to occur in the near future. The LCM used in this demonstration is capable of projecting such changes, although limited GIS data on minor road and canal networks precluded this sort of analysis in this study. However, experimentation with the LCM used here in other contexts [34] suggests that parameterizing the growth of linear networks can prove very challenging.

Although it was originally hypothesized that climate layers would enhance the SDM results, low average p(***s***) values for test points and anomalously high p(***s***) values in hyperarid areas far from major rivers indicated that inclusion of climate data produced unrealistic SDM outputs. In light of the important invasion event in the 1940s when the species must have been widespread in the Nile Valley of Egypt, it is concluded that temperature seasonality and isothermality are not limiting factors anywhere in the Nile Valley and that rainfall is generally too low to support establishment in Upper Egypt. Therefore, irrigation as evidenced by cropland and NDVI was used as the main variable that would affect future distribution of the vector. This result also suggests that at this scale of investigation (as shown in Figure1), 1 km climate data are unlikely to improve SDM projections and may even worsen them if applied to regions that do not possess major climate gradients.

Irrigation from surface and ground water is likely to continue to be a major driver of environmental change in North Africa and the Middle East in order to meet growing regional and global demand for food and realize export potential. Moreover, other tropical regions within South Asia, Sub-Saharan Africa and Latin America are likely to experience expansion of irrigation infrastructure as well in the coming decades [20]. Beyond irrigation, deforestation for agricultural expansion is another major concern wherein LCMs and SDMs may be linked, particularly where forest is being transformed into pastures and farmland in the Amazon Basin. Additional applications involving LCMs and SDMs include habitat fragmentation, urbanization, road building, and wetland modification, to name but a few [13]. However, while LCMs have potential to project land cover changes associated with these processes, these applications within the context of vector-borne disease studies remain largely underexploited.

While this study provides a new methodological demonstration, several limitations are worth noting, including the assumption of constant transition probability inherent in the Markov Chain approach, which is common to many LCMs [34], the assumption that the occurrence data used here can be used to represent future distribution (i.e. that presence records are static) and therefore may be used to evaluate the results of distribution projections decades into the future, and the limited availability of vector point data to parameterize SDMs in areas such as North Africa. Areas of future research may include a range of different land change and species distribution models to explore further the variability of LCM and SDM outputs in different contexts. The results of such investigations may be useful for deterministic models that evaluate pathogen transmission and epidemic potential within and between homogeneous and non-homogeneous habitat patches and help to guide more realistic model formulation by accounting for multiple patches of variable size and inter-patch distances. Further, the approach used here may be routinely applied to assess potential impacts of future irrigation/agricultural development projects in arid areas. Most countries currently have legislation ensuring that future irrigation development projects are subject to environmental assessments and, therefore, linking LCMs and SDMs can assist countries in managing negative impacts related to vectors and vector-borne diseases in conjunction with other data and tools employed in environmental and disease impact assessments.

## Competing interests

The authors declare that they have no competing interests.

## Authors’ contributions

DOF conceived and wrote the paper as well as conducted the modeling, MP processed the environmental and other GIS data layers used in both models and contributed to the writing, ANH provided GIS data and *Anopheles* distribution information and helped to write the paper. JCB helped to conceive and write the paper. All authors have read and approved the final version.
